# Morphological, Optical, and Electrical Properties of p-Type Nickel Oxide Thin Films by Nonvacuum Deposition

**DOI:** 10.3390/nano10040636

**Published:** 2020-03-29

**Authors:** Chien-Chen Diao, Chun-Yuan Huang, Cheng-Fu Yang, Chia-Ching Wu

**Affiliations:** 1 Department of Electronic Engineering, Kao Yuan University, Kaohsiung 821, Taiwan; ccd@kyu.edu.com; 2 Department of Applied Science, National Taitung University, Taitung 950, Taiwan; laputa@nttu.edu.tw; 3 Department of Chemical and Materials Engineering, National University of Kaohsiung, Kaohsiung 811, Taiwan

**Keywords:** lithium-doped nickel oxide, non-vacuum deposition, figure of merit, heterojunction diode

## Abstract

In this study, a p-type 2 at% lithium-doped nickel oxide (abbreviation L2NiO) solution was prepared using Ni(NO_3_)_2_·6H_2_O, and LiNO_3_·L2NiO thin films were deposited using an atomizer by spraying the L2NiO solution onto a glass substrate. The sprayed specimen was heated at a low temperature (140 °C) and annealed at different high temperatures and times. This method can reduce the evaporation ratio of the L2NiO solution, affording high-order nucleating points on the substrate. The L2NiO thin films were characterized by X-ray diffraction, scanning electron microscopy, UV–visible spectroscopy, and electrical properties. The figure of merit (FOM) for L2NiO thin films was calculated by Haacke’s formula, and the maximum value was found to be 5.3 × 10^−6^ Ω^−1^. FOM results revealed that the L2NiO thin films annealed at 600 °C for 3 h exhibited satisfactory optical and electrical characteristics for photoelectric device applications. Finally, a transparent heterojunction diode was successfully prepared using the L2NiO/indium tin oxide (ITO) structure. The current–voltage characteristics revealed that the transparent heterojunction diode exhibited rectifying properties, with a turn-on voltage of 1.04 V, a leakage current of 1.09 × 10^−4^ A/cm^2^ (at 1.1 V), and an ideality factor of *n* = 0.46.

## 1. Introduction

**Figure 1:**
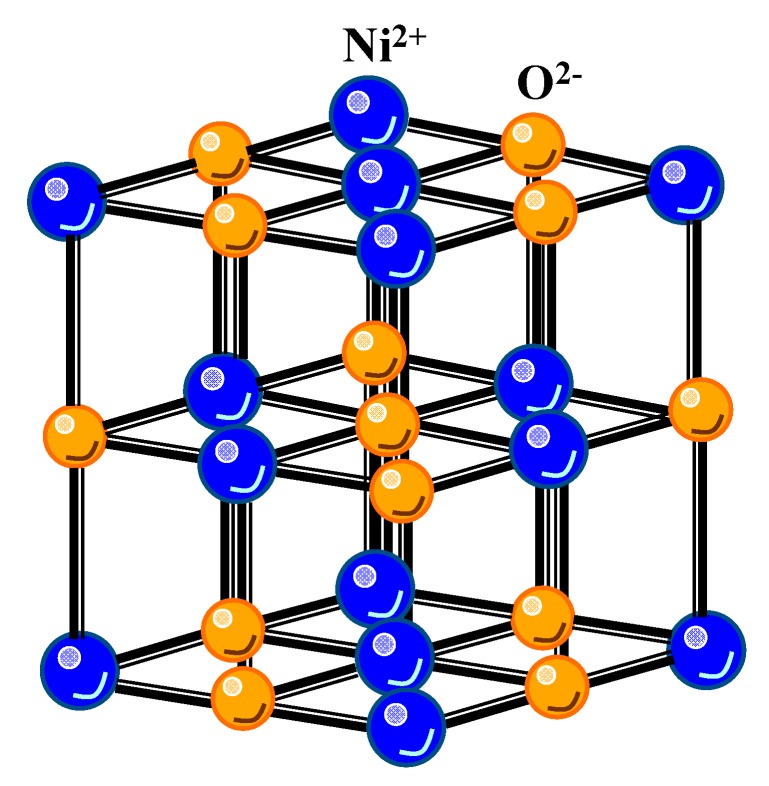
Crystal structure of the NiO thin film.

At present, numerous applications, such as touch panels, light-emitting diodes, and solar cells, require transparent, conductive coatings [1,2,3]. Thus far, materials belonging to the transparent conducting oxide (TCO) family have been frequently used for this purpose. Most of the industry standard TCO are n-type wide bandgap oxides (Eg > 3.1 eV), such as In_2_O_3_, SnO_2_, and ZnO, whose conductivity can be further tuned by aliovalent doping or the formation of oxygen vacancies [4]. In contrast, the development of p-type TOS remains a challenge. Recently, semi-transparent p-type conducting films of the nickel oxide (NiO) have attracted considerable attention because of their importance in several scientific applications, including (i)material for electrochromic display devices [5,6], (ii) functional sensor layers in chemical sensors [7], (iii) transparent electronic devices [8] and (iv) the magnetic properties of nanoparticles [9,10,11,12]. A stoichiometric NiO thin film is an insulator at room temperature (resistivity is ~10^13^ Ω·cm) [13]. Much effort has been made to explain the insulating behavior of NiO. NiO crystallizes in a rock-salt crystal structure, in which Ni cations have a nominal valence state of 2+ (3d^8^) in octahedral coordination (see Figure 1). Due to a strong electron correlation in 3d orbitals, it has an optical bandgap of 3.4–4.0 eV [14]. In addition, according to the literature, at temperatures above the Néel temperature (523 K), the crystal structure of NiO is cubic, whereas below the Néel temperature, the crystals become slightly distorted and acquire a rhombohedral structure which accompanies the antiferromagnetic ordering [15].

NiO thin films can be grown by several chemical and physical methods, including magnetron sputtering [16,17], evaporation [18], the sol–gel method [19], laser ablation deposition [20], and spray pyrolysis (SP) [21,22]. NiO thin films with low resistivity (1.4 × 10^−1^ Ω·cm) can be deposited by sputtering [23]. Compared with vacuum deposition, SP is a relatively simple, cost-effective nonvacuum deposition method for fabricating TCO thin films for large-area coating. However, the resistivity of the doped NiO thin films fabricated by SP is ~10^4^ Ω·cm [24]; this resistivity is several orders of magnitude greater than that observed for sputter-deposited NiO thin films. Conventional SP involves spraying a nickel nitrate solution onto a preheated glass substrate at a temperature greater than 300 °C, followed by evaporation, solute precipitation, and pyrolytic decomposition. With the increase in the substrate temperature, the evaporation ratio of the solution on the substrate is extremely swift, leading to the formation of inferior NiO thin films. To solve this problem, a modified spray method was used in this study. First, the substrate temperature is slightly greater than the boiling point of the spray solution. The evaporation ratio of the spray solution decreases, affording high-order nucleation points by the spraying of the solution onto the substrate. The thin films were then formed and further annealed at high temperatures to afford a crystalline structure. Finally, high-quality thin films were obtained and subsequently applied as a photoelectric device.

To improve the conductivity of the NiO thin film, three improved mechanisms were used: (i) holes generated from nickel vacancies, (ii) oxygen interstitial atoms, and (iii) monovalent atoms used as a dopant. Monovalent atoms can be used as the dopant to increase the electrical conductivity of the NiO thin films [25,26]. In this study, a modified spray method was employed for the deposition of 2 at% Lithium (Li)-doped NiO (L2NiO) thin films with a high electrical conductivity. The monovalent atoms of Li can be substitution Ni atoms. In addition, the effects of annealing temperatures and times on the physical, optical, and electrical properties of the L2NiO thin films were investigated. X-ray photoelectron spectroscopy (XPS) was used to investigate the variations in the characteristics of the L2NiO thin films. Finally, a transparent heterojunction diode device comprising an L2NiO thin film and an indium–tin oxide (ITO) thin film was fabricated for future applications.

## 2. Experimental Methods 

Lithium-doped nickel oxide (LNiO) thin films were deposited on a Corning glass substrate by the modified spray method. The spray solution was prepared by mixing nickel nitrate (Ni(NO_3_)_2_·6H_2_O, Alfa Aesar, MA, USA) and lithium nitrate (LiNO_3_, J.T. Baker, NJ, USA) in deionized (DI) water. A 1 M L2NiO spray solution was prepared by doping 2 at% Li in NiO. The modified spray method involved spraying a L2NiO solution at 140 °C, which then evaporated, affording high-quality L2NiO thin films on the Corning glass substrate. The L2NiO thin films were deposited under the following conditions: solution volume = 40 mL, deposition rate = 10 mL/min. The distance between the Corning glass substrate and the nozzle was approximately 20 cm, and compressed air was used as the carrier gas. Annealing temperatures and times were 400–600 °C and 1–3 h, respectively, for the crystallization of the L2NiO thin films. Finally, to fabricate the transparent heterojunction diodes, L2NiO thin films were deposited on an ITO glass substrate, and the top and bottom aluminum (Al) electrodes were deposited by electron-beam evaporation. The surface morphology of the L2NiO thin films were examined by high-resolution scanning electron microscopy (HR-SEM, Hitachi, Japan). The resulting interface layer morphology between the L2NiO and ITO thin film was characterized by high-resolution transmission electron microscopy (HR-TEM, JOEL, Japan). The phase and crystallinity of the L2NiO thin films were measured by X-ray diffraction (XRD, Bruker, MA, USA) using CuKα radiation in the 2θ range of 20°–80°. The bonding state and element content of the L2NiO thin films were investigated using X-ray photoemission spectroscopy (XPS, ULVAC·PHI, Japan). The XPS using a monochromatic Al Kα X-ray (hν = 1486.6 eV) source was carried out at normal emission with an electron energy analyzer. The resistivity, carrier concentration, and mobility were measured by Hall effect measurements using the Van der Pauw method. The optical transmittance of the L2NiO thin films was measured using a UV–vis system (Agilent, CA, USA), and the transmittance spectrum was recorded as a function of the wavelength in the range of 200 to 1100 nm. The current–voltage (I–V) properties of the transparent heterojunction diode was measured using an HP4156 semiconductor parameter analyzer (Agilent, CA, USA).

## 3. Results and Discussion

**Figure 2:**
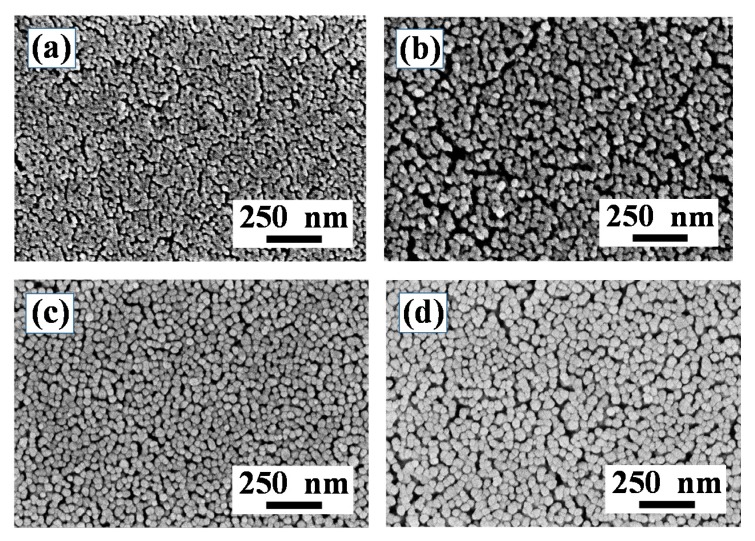
Surface SEM images of the L2NiO thin films as a function of annealing temperatures and times: (**a**) 400 °C for 1 h, (**b**) 400 °C for 3 h, (**c**) 500 °C for 3 h, and (**d**) 600 °C for 3 h.

Figure 2 shows the HR-SEM images of the L2NiO thin film with different annealing temperatures and times. The HR-SEM image of the L2NiO thin film which had annealed at 400 °C for 1 h revealed a smooth surface and no grain growth (Figure 2a). With the further increase in the annealing time to 3 h at 400 °C, the surface morphology revealed small grain sizes (Figure 2b). The average grain size of the film annealed at 400 °C for 3 h was 38 nm. Surface SEM morphologies shown in Figure 2c,d were compared by the increase in the annealing temperature of the L2NiO thin films from 500 °C to 600 °C for 3 h, and the grain sizes slightly increased. At annealing temperatures of 500 °C and 600 °C for 3 h, the average grain sizes of the L2NiO thin films were 45 nm and 58 nm, respectively. As a result of annealing at higher temperatures, the surface atoms on the substrate acquire more energy, and these atoms can move to suitable nucleation sites. In addition, the low activation energy ions doped in the thin film can easily escape from trap sites and transfer to nucleation sites. Crystalline thin films can be obtained when an increased number of better nucleation sites are formed on the substrate. In this study, the low activation energy of the Li ions doped in the NiO thin film leads to the increase in grain size with the increase in the annealing temperatures and times [27]. Compared with previous reports, the crystalline grain structure of the L2NiO thin films deposited by the modified spray method is better than that obtained by SP [28,29], because SP involves the deposition of the solution onto a preheated (>300 °C) substrate, but the evaporation ratio of the solution is extremely swift, affording poor nucleating points. Therefore, the surface morphology of the thin film is not good. The thickness of the L2NiO thin film with different annealing temperatures and times is shown in cross-section SEM images (Figure S1). The thickness of the L2NiO thin film annealed at 400 °C for 1 h was 202 nm. The thickness of the L2NiO thin films increased slightly as the annealing temperatures and times increased.

**Figure 3:**
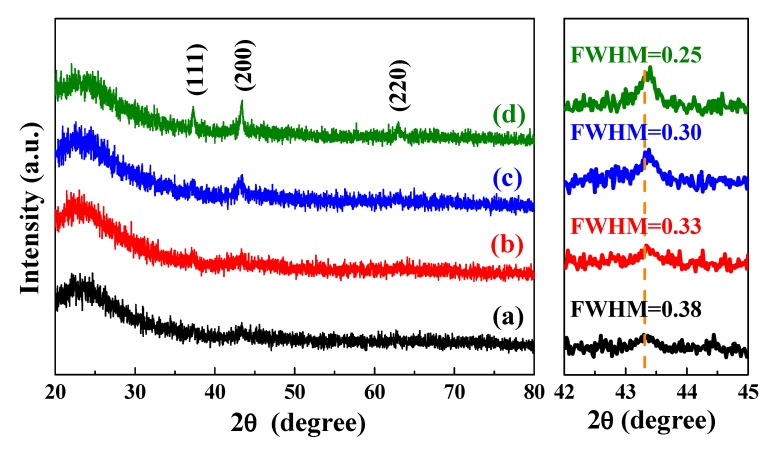
X-ray diffraction patterns of the L2NiO thin films as a function of the annealing temperatures and times: (**a**) 400°C for 1 h, (**b**) 400°C for 3 h, (**c**) 500°C for 3 h, and (**d**) 600°C for 3 h.

The crystalline structure of the L2NiO thin films was examined by XRD using CuKα (λ = 0.1542 nm) radiation. Figure 3 shows the XRD patterns of the L2NiO thin films with different annealing temperatures and times. The observed XRD patterns of the L2NiO thin films were compared with the Joint Committee on Powder Diffraction Standards (JCPDS) data; they were in good agreement with the standard diffraction pattern of NiO (JCPDS card no. 47-1049). Diffraction peaks for the L2NiO thin films were observed at 2θ values of 37.3°, 43.2°, and 63.1°, which correspond to the (111), (200), and (220) planes, respectively. The L2NiO thin films were polycrystalline without any other detectable secondary phase. Diffraction results revealed that the L2NiO thin film annealed at 400 °C for 1 h exhibited an approximate amorphous structure due to its weak-intensity diffraction peaks (Figure 3a). However, with the increase in the annealing temperatures and times from 400 °C to 600 °C and 1 to 3 h, respectively, diffraction intensities for the (111), (200), and (220) planes slightly increased (Figure 3b–d). The increase in the diffraction intensity was related to the grain sizes of the L2NiO thin films. Figure 3 (right side) also shows the grazing incidence angle X-ray diffraction patterns (GIAXRD) of the L2NiO films in the 2θ range of 42° to 45°. The full-width half-maximum for the diffraction peak of the (200) plane of the L2NiO thin films decreased from 0.38 to 0.25. The crystallite size of the L2NiO thin films was then calculated using the Scherrer equation. With the increase in the annealing temperatures and times, the grain sizes increased from 39 nm to 60 nm. The results obtained for the various grain sizes were similar to those from SEM (Figure 2). In addition, with the increase in the annealing temperatures and times, the (200) plane was slightly shifted to high angles. According to Bragg’s law (*nλ =* 2*dsinθ*) and *d = a/(h^2^ + k^2^ + l^2^)*^1/2^, the lattice constant (a) slightly decreased from 4.178 Å to 4.169 Å with the increase in the annealing temperatures and times, indicating that the larger radius of Ni^2+^ (0.69 Å) can be substituted by the smaller radius of Li^+^ (0.68 Å); this subsequently leads to the decreased lattice constant of L2NiO thin films [30]. 

**Figure 4:**
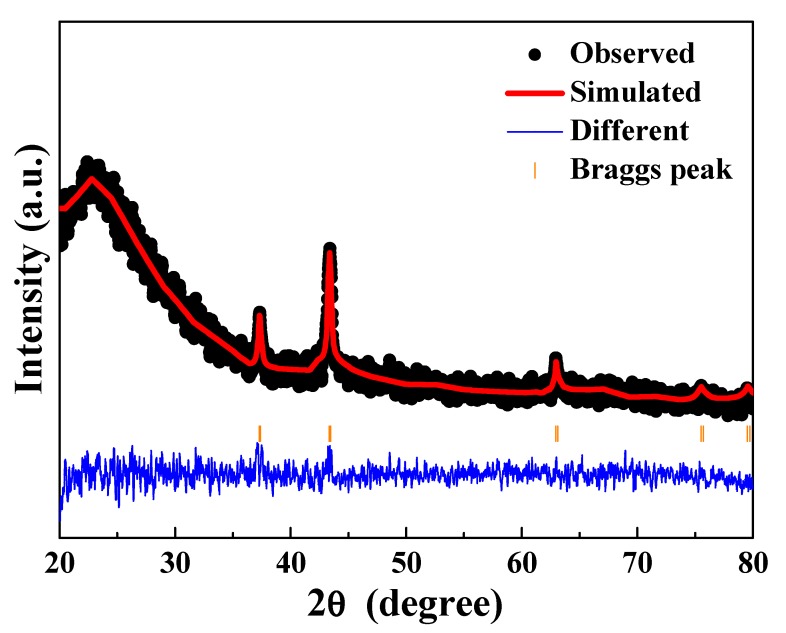
Rietveld refinement of the L2NiO thin films produced with an annealing temperature of 600°C for 3 h.

The crystal structure parameter of the L2NiO thin films produced with a 600 °C annealing temperature for 3h was fitted using the cubic structural model, with the atomic positions being described in the space group Fm3m. The fitted profiles of the L2NiO thin films for XRD data at 600 °C annealing temperature for 3h is shown in Figure 4. The final refinement convergence of the L2NiO thin films produced with a 600 °C annealing temperature for 3h was achieved with χ^2^ = 1.38, and the measured result agreed well with the simulation value. i.e., the lattice constant (a) of the L2NiO thin was 4.1686 Å, similar to the value calculated using Bragg’s law. The refined values of all thin film are also tabulated in Table 1.

**Figure 5:**
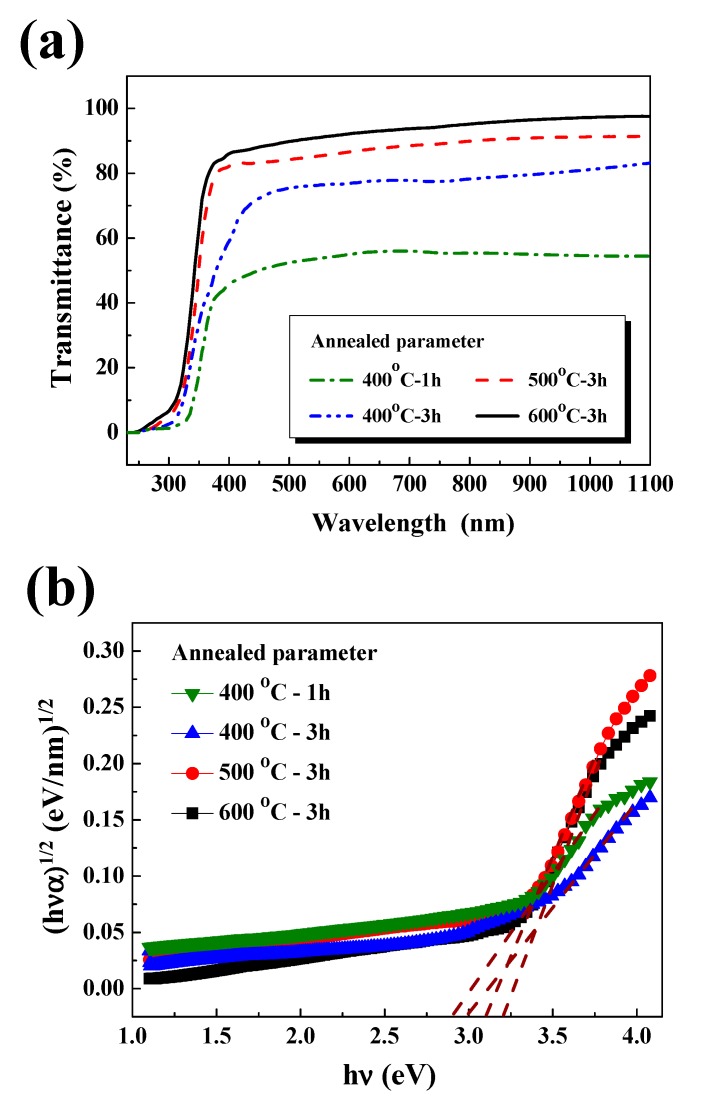
(**a**) Optical transmittance spectra and (**b**) optical bandgap of the L2NiO thin films as a function of annealing temperatures and times.

Figure 5a shows the optical transmittance spectra of the L2NiO thin films in the 250–1100 nm range. For the L2NiO thin films annealed at 400 °C for 1 h and those annealed at 400 °C, 500 °C, and 600 °C for 3 h, average transmittance values in the visible region (400 to 700 nm) were 46.8%, 72.3%, 84.6%, and 87.9%, respectively. The increase in the average transmittance of the L2NiO thin films was related to the increase in the grain size and decrease in the grain boundary, leading to the low scattering effect in L2NiO thin films. Surface SEM images revealed that the grain size of the L2NiO thin films increased with different annealing temperatures and times; this result was in agreement with the optical transmittance results. In the ultraviolet range, with the increase in the annealing temperature from 400 °C to 600 °C at an annealing time of 1 h to 3 h, the absorption edge was shifted toward a short wavelength region. The blue-shift can be explained by the Burstein–Moss shift effect [32,33,34].

The optical energy band gap (E_g_) is an important physical parameter that mainly determines the electrical and optical characteristics of materials. The energy band gap of the L2NiO thin films were determined by applying the Tauc and Davis-Mott models [35]:

(1) $${\left(\alpha h\nu \right)}^{1/2}=B\left(h\nu -E_{g}^{op}\right)$$

where *α* is the absorption coefficient, h is Planck’s constant, ν is the frequency of the incident photon, and *B* is the absorption edge width. The energy band gap was determined by the extrapolation of a straight linear region of the plots to *hν* = 0 [36]. Figure 5b shows (*αhν*)^1/2^ vs. *hν* for L2NiO thin films with different annealing temperatures and times. With the increase in the annealing temperatures from 400 °C to 600 °C and the annealing times from 1 h to 3 h, the energy band gap of the L2NiO thin films increased from 2.89 to 3.21 eV (Figure 5b), suggesting that the Burstein–Moss effect may affect the band gap shift [33,34]. In the Burstein–Moss effect, the band-gap shift is mainly related to the high carrier concentration and/or low effective mass. According to the Burstein–Moss effect, the divergence of the band gap is expressed as

(2) $$\Delta E_{g}^{BM}=\frac{h^{2}}{2m_{vc}^{*}}{\left(3\pi^{2}n\right)}^{\frac{2}{3}}$$

where Δ*Eg^BM^* is the shift value between the doped semiconductor and undoped semiconductor, m_cv_^*^ is the reduced effective mass, and n is the carrier concentration. The absorption edge of the L2NiO thin films were observed in a shorter wavelength region because of the increase in the carrier concentration (*n*) (Figure 5a).

**Figure 6:**
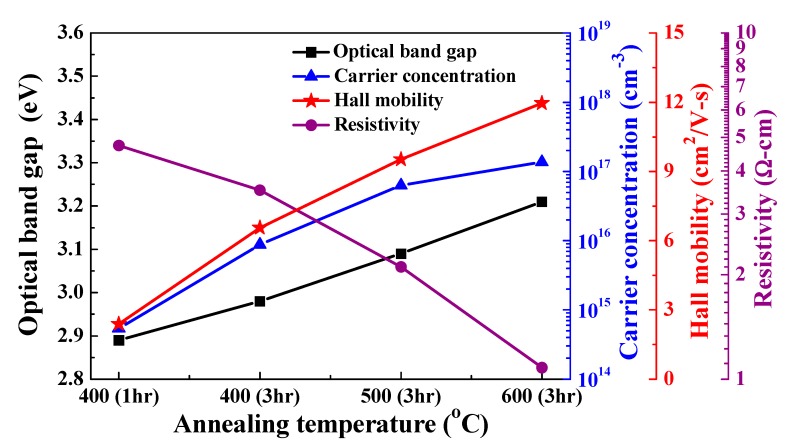
Optical band gap, carrier concentration, mobility, and resistivity of the L2NiO thin films as a function of annealing temperatures and times.

Figure 6 shows the optical energy band gap, carrier concentration (*n*), mobility (*μ*), and resistivity (*ρ*) of the L2NiO thin films with different annealing temperatures and times. All samples exhibited p-type properties. With the increase in the annealing temperatures and times, the mobility of the L2NiO thin films increased from 2.39 to 11.96 cm^2^/Vs because of the increase in the grain size and decrease in the grain boundary, causing the carrier to encounter less hindering materials, and subsequently resulting in increased carrier mobility. Meanwhile, the increase in the annealing temperatures and times caused an increase in the carrier concentration. The number of Li atoms substituting the sites of Ni atoms increased, leading to the substitution of a large number of Ni^2+^ by Li ions in the normal crystal sites and creating holes at a high annealing temperature. Therefore, the carrier concentration of the L2NiO thin films increased; this result can be obtained by Equation (3).

(3) $$\frac{1}{2}O_{2}^{\left(g\right)}{+\mathrm{Li}}_{2}O\underset{}{\Leftrightarrow }{2O}_{x}^{O}{+2\mathrm{Li}}_{Ni\prime }+2\;\overset{\cdot }{h}$$

With the increase in the annealing temperatures and times, the Li concentration of the L2NiO thin films increased, as demonstrated by the XPS analysis shown in Table 2 and the XRD analysis shown in Figure 3 (right side). The resistivity of the film is known to be proportional to the reciprocal of the product of carrier concentration and mobility, as follows:

*ρ* = 1 / (*n* × *μ*) (4)

Therefore, with the increase in the carrier concentration and mobility, the resistivity of the L2NiO thin films decreased from 4.73 to 1.08 Ω·cm. Compared with previous reports, the resistivity of L2NiO thin films was slightly less than those of undoped NiO thin films [36,37].

**Figure 7:**
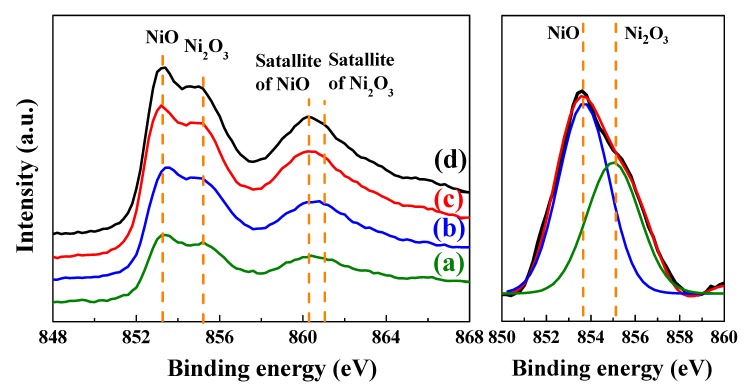
Ni 2p_3/2_ XPS spectra of the L2NiO thin films as a function of annealing temperatures and times: (**a**) 400°C for 1 h, (**b**) 400°C for 3 h, (**c**) 500°C for 3 h, and (**d**) 600°C for 3 h.

Figure 7 shows the Ni 2p_3/2_ XPS spectra of the L2NiO thin films with different annealing temperatures and times. During the Gaussian fitting process, binding energies for the NiO and Ni_2_O_3_ peaks were observed at 854.0 eV and 855.8 eV, respectively, in the Ni 2p_3/2_ XPS spectra of the L2NiO thin films annealed at 400 °C for 1 h (right side of Figure 7) [38,39]. The Ni_2_O_3_ and NiO peaks corresponded to Ni^3+^ and Ni^2+^, respectively. With the increase in annealing temperatures and times for the L2NiO thin films, the intensity of the Ni^3+^ bonding state slightly increased over that of the Ni^2+^ bonding state. This result is related to the insertion of an excess amount of oxygen ions in the interstitial sites of the L2NiO thin films due to annealing conducted in the atmosphere; this leads to the formation of Ni^3+^ ions and holes, which can be represented by Equation (5).

(5) $$\frac{1}{2}O_{2}^{\left(g\right)}\underset{}{\Leftrightarrow }O_{i}^{\prime \prime }+2\;\overset{\cdot }{h}$$

**Figure 8:**
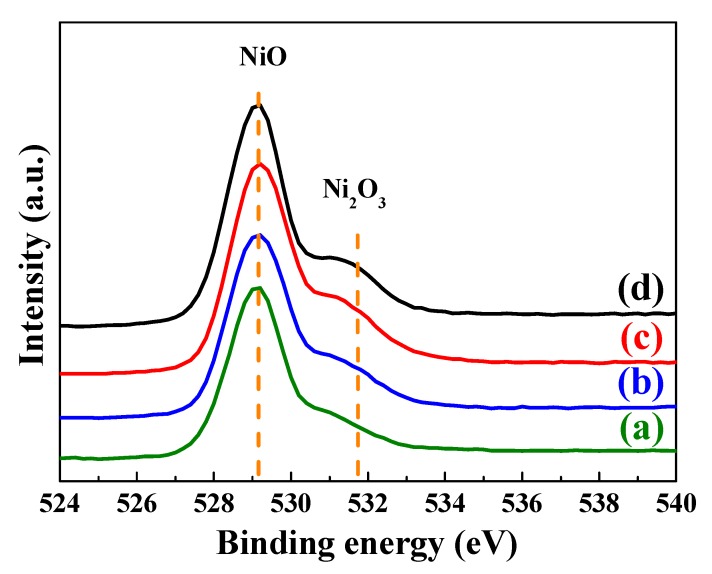
O 1s XPS spectra of the L2NiO thin films as a function of annealing temperatures and times. (**a**) 400°C for 1 h, (**b**) 400°C for 3 h, (**c**) 500°C for 3 h, and (**d**) 600°C for 3 h.

Meanwhile, the Ni 2p_3/2_ results were also confirmed from the O1s XPS spectra of the L2NiO thin films with annealing temperatures and times, as shown in Figure 8. With the increase in the annealing temperature and times, the intensity of the O1s peak increased appreciably. In the O1s XPS spectra, the deconvolution of the electron binding energy of NiO (529.3 eV) and Ni_2_O_3_ (531.7 eV) was observed for the L2NiO thin films [38,40,41]. The increase in the magnitude of the Ni^3+^ bonding state was slightly greater than that of the Ni^2+^ bonding state, demonstrating that the hole carrier concentration of the L2NiO thin films increased with the annealing temperatures and times. The increased hole carrier concentration of the L2NiO thin films was in agreement with the Hall measurement (Figure 6).

**Figure 9:**
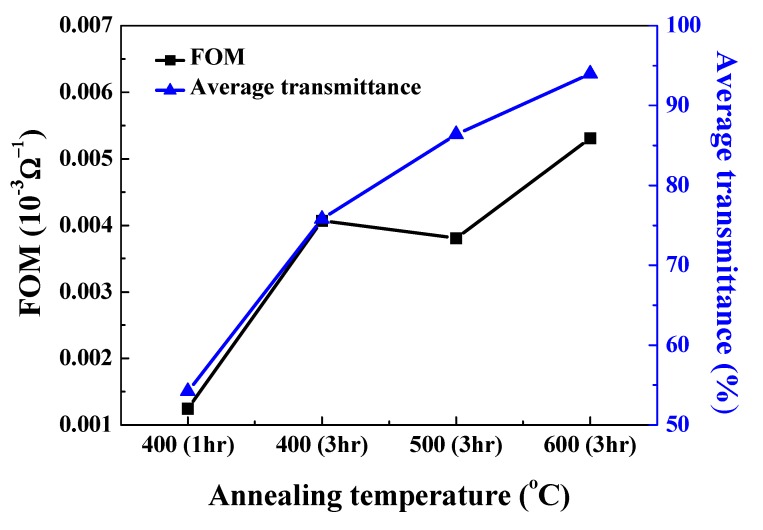
FOM values for L2NiO thin films as a function of annealing temperatures and time.

Figure 9 shows the figure-of-merit (FOM) of the L2NiO thin films with different annealing temperatures and times. To deposit L2NiO thin films with high transmission and low resistivity, the FOM values for the L2NiO thin films with different annealing temperatures and times were calculated by using Haacke’s equation [42]:

(6) $$\mathrm{FOM}=\frac{T^{10}}{R_{s}}$$

where *T* is the average optical transmittance at 400–700 nm and *R_s_* is the sheet resistance of the L2NiO thin films. With the increase in annealing temperatures and times, the FOM of the L2NiO thin films increased (Figure 9). The maximum FOM (5.3 × 10^−6^ Ω^−1^) was obtained for the L2NiO thin films annealed at 600 °C for 3 h. FOM results revealed that L2NiO thin films exhibit satisfactory optical and electrical characteristics for photoelectric device applications.

**Figure 10:**
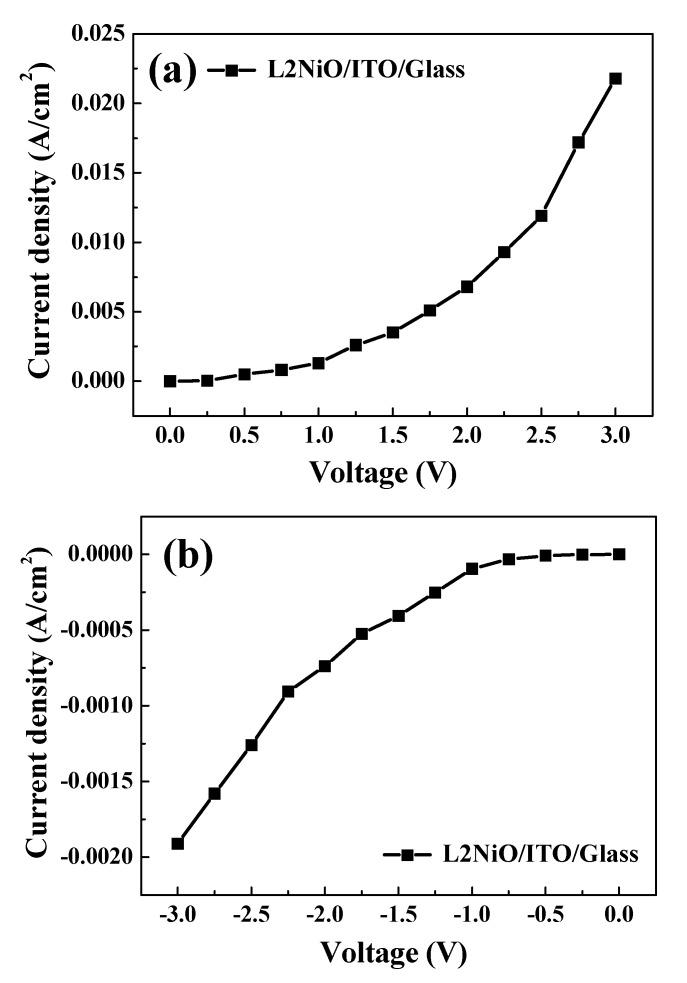
Current–voltage curve of the L2NiO/ITO transparent heterojunction diode: (**a**) forward current and (**b**) reverse current.

From the above results, it can be seen that the carrier concentration, mobility, and conductivity characteristics of the Li ions doped in NiO thin film improved compared to non-doped NiO thin film. For the possible fabrication of transparent heterojunction diodes, the L2NiO thin films were then deposited at an annealing temperature of 600 °C and an annealing time of 3 h onto an ITO glass substrate to form a p–n junction structure. The ITO thin film was deposited by the sputtering method. The thickness of the ITO thin film was 1000 Å with a 92% visible-light transmittance and a resistance of 12 Ω·cm, as shown in Figure S2a,b. Figure 10 shows the current–voltage (I–V) curve of the L2NiO/ITO transparent heterojunction diode. The I–V results confirmed that the fabricated L2NiO/ITO transparent heterojunction diode exhibited rectifying behavior with the use of aluminum (Al) as the electrodes. Before the measurement of the I–V properties of the L2NiO/ITO transparent heterojunction diode, ohmic contacts were confirmed to be present between the Al electrodes and the L2NiO and ITO thin films. Under forward bias, the turn-on voltage for the L2NiO/ITO transparent heterojunction diode was ~1.04 V (Figure 10a); this value is less than that (2.57 V) for a p-NiO/n-TZO diode [43], 2.5 V for a p-CuO/n-ZnO diode [44], and similar (1 V) to that for a p-NiO/n-ZnO diode [45]. Under a reverse voltage, the leakage current was 1.09 × 10^−4^ A/cm^2^ at 1.1 V for the L2NiO/ITO transparent heterojunction diode (Figure 10b). The rectification ratio (R) for the L2NiO/ITO transparent heterojunction diodes was calculated using Equation (7) as follows to obtain a value of 17.3 (at 1.1 V):

(7) $$R=\frac{\mathrm{Forward}\;\mathrm{current}}{\mathrm{Reverse}\;\mathrm{current}}$$

**Figure 11:**
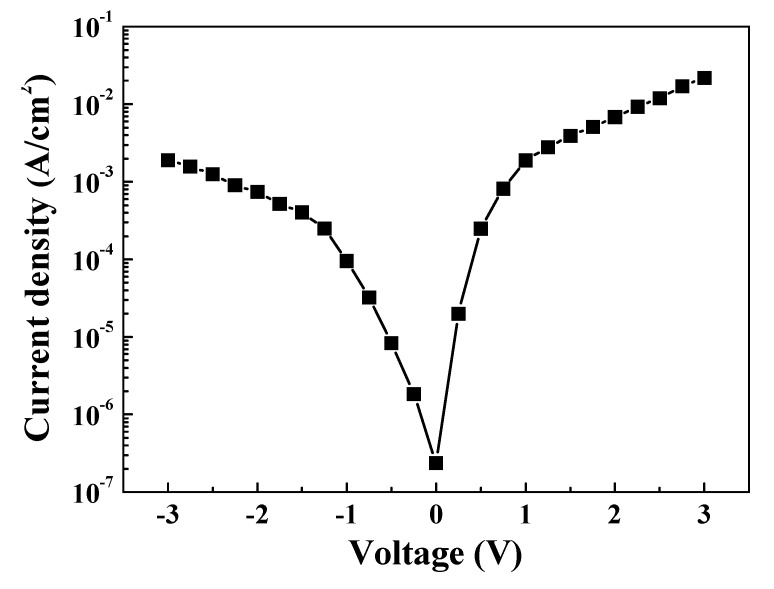
Log current–voltage curve of the L2NiO/ITO transparent heterojunction diode.

The ideality factor (*n*) can be calculated from the slope of the linear region of the forward-bias log(I)–V curve, which can be derived from Equation (8) and Figure 11:

(8) $$n=\frac{q}{kT}\times \left[\frac{dV}{dln\left(I\right)}\right]$$

where *k* is the Boltzmann constant, *T* is the temperature in kelvin, and *q* is the electron charge. The ideality factor for the L2NiO/ITO transparent heterojunction diode was *n* = 0.46, which was less than the ideal value of *n* = 1 (Equation (8)). The high leakage current and low ideality factor result from imperfections between the heterojunction interfaces of the L2NiO and ITO thin films. Ajimsha et al. reported that in an oxide layer, these imperfections were caused by the presence of different crystal-type structures [46].

**Figure 12:**
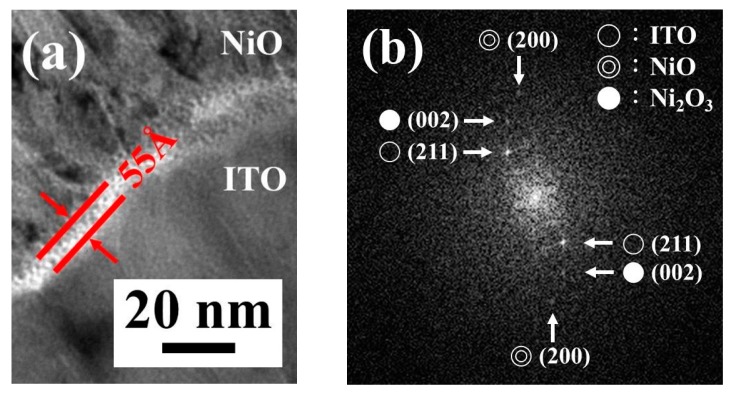
(a) TEM image and (b) SAED pattern of the L2NiO/ITO transparent heterojunction structure.

To further investigate the interface between the L2NiO and ITO thin films, TEM images were recorded. Figure 12a shows a magnified TEM image of the interface between the L2NiO thin film annealed at 600 °C for 3 h and the ITO thin film; a clear layer was present at the interface between the two materials. The interfacial layer thickness was 55 Å; this layer was thought to be NiO_2_, Ni_2_O_3_, and Ni_3_O_4_, because during spraying, Ni and O can be easily combined with In or Sn in ITO. This result can be attributed to the relatively high bond energy of Ni–O (1029 kJ/mol) compared with those of Sn–O (531.8 kJ/mol) and In–O (320 kJ/mol) [47,48,49]. From the XPS result, it was hypothesized that the interfacial layer between the NiO and ITO was Ni_2_O_3_. Figure 12b shows the selected-area electron diffraction (SAED) pattern of the boundary between the L2NiO and ITO thin films. The corresponding SAED pattern exhibited (200) NiO, (211) ITO, and (002) Ni_2_O_3_ diffraction rings, confirming that the L2NiO (including NiO and Ni_2_O_3_) and ITO thin films are polycrystalline; this result also confirmed that a thin Ni_2_O_3_ interfacial layer was present between the L2NiO and ITO thin films. The thin Ni_2_O_3_ interfacial oxide layer rendered a high leakage current and low ideality factor for the L2NiO/ITO transparent heterojunction diode.

## 4. Conclusions

In this study, a modified spray method was used for the deposition of high-quality 2 % Li-doped NiO (L2NiO) thin films. The L2NiO thin films exhibited a cubic (NaCl-type) structure, and the lattice constant of the L2NiO thin films slightly decreased from 0.4178 Å to 0.4169 Å with the increase in annealing temperatures and times. As the smaller radius of Li^+^ (0.6 Å) was substituted by the larger Ni^2+^ (0.69 Å), the number of substituted Li^+^ increased, leading to a decrease in the lattice constant of the L2NiO thin films. According to the Burstein–Moss shift theory, the optical energy band gap (E_g_) of the L2NiO thin films increased from 2.89 eV to 3.21 eV with the increase in the annealing temperatures and times because of the increase in the carrier concentration. In Hall measurements, the carrier concentration and mobility of the L2NiO thin films increased, leading to a decrease in the resistivity from 4.73 Ω·cm to 1.08 Ω·cm with the increase in the annealing temperatures and times. The optimum FOM (5.3 × 10^−6^ Ω^−1^) was obtained for the L2NiO thin films annealed at 600 °C for 3 h, for which the resistivity and average transmittance were 1.08 Ω·cm and 87.9%, respectively. Finally, the transparent heterojunction diode comprising a p-type L2NiO thin film and an n-type ITO thin film was successfully fabricated. Its properties included (1) a turn-on voltage of 1.04 V, (2) a leakage current of 1.09 × 10^−4^ A/cm^2^ (at 1.1 V), (3) a rectification ratio of 17.3, and (4) an ideality factor of 0.46. The high leakage current resulted from the Ni_2_O_3_ thin layer between the heterojunction interfaces of the different crystal-type structure with the L2NiO and ITO thin films. Therefore, the L2NiO film was shown to possess satisfactory properties for applications including transparent diode, electrochromic display, and solar cell devices.

## Figures and Tables

**Table 1:** Refined values of the L2NiO thin films with different annealing temperatures and times.

**Parameter**	Nondoped NiO (R [31])	L2NiO (400°C, 1 h)	L2NiO (400°C, 3 h)	L2NiO (500°C, 3 h)	L2NiO (600°C, 3 h)
a = c = b (Å)	4.1801	4.1774	4.1738	4.1701	4.1686
α = β = γ	90°	90°	90°	90°	90°
Volume (Å^3^)	73.01	72.49	72.44	72.41	72.38

**Table 2:** Elements of L2NiO thin films as a function of annealing temperature and time.

	Ni (at%)	O (at%)	Li (at%)	O/Ni
L2NiO-400°C-1 h	45.23	52.96	1.81	1.171
L2NiO-400°C-3 h	45.1	53.07	1.83	1.176
L2NiO-500°C-4 h	44.9	53.23	1.87	1.185
L2NiO-600°C-3 h	44.62	53.50	1.88	1.199

## Supplementary Materials

The following are available online at https://www.mdpi.com/2079-4991/10/4/636/s1, Figure S1: Cross-section SEM images of the L2NiO thin films as a function of annealing temperatures and times: (a) 400 °C for 1 h, (b) 400 °C for 3 h, (c) 500 °C for 3 h, and (d) 600 °C for 3 h. Figure S2: (a) Cross-section SEM image and (b) optical transmittance spectra of the ITO thin film.
