# Supplementary material for: Morphological, Optical, and Electrical Properties of p-Type Nickel Oxide Thin Films by Nonvacuum Deposition
Source: Nanomaterials (Basel). 2020 Mar 29;10(4):636. doi: 10.3390/nano10040636 (PMC7221566; doi:10.3390/nano10040636)
Supplement: Supplementary file 1 [file nanomaterials-10-00636-s001.pdf]

Supplementary file

Morphological, Optical, and Electrical Properties of p-type Nickel Oxide Thin Films by Non-Vacuum Deposition

Chien-Chen Diao[1], Chun-Yuan Huang[2], Cheng-Fu Yang[3,*], Chia-Ching Wu[2,*]

1. Department of Electronic Engineering, Kao Yuan University, Kaohsiung 821, Taiwan; ccd@kyu.edu.com (C.C.D.)
2. Department of Applied Science, National Taitung University, Taitung 950, Taiwan; laputa@nttu.edu.tw (C.Y.H.); ccwu@nttu.edu.tw (C.C.W.)
3. Department of Chemical and Materials Engineering, National University of Kaohsiung, Kaohsiung 811, Taiwan; cfyang@nku.edu.com (C.F.Y.)

* Correspondence: cfyang@nku.edu.com (C.F.Y.) and ccwu@nttu.edu.tw (C.C.W.)

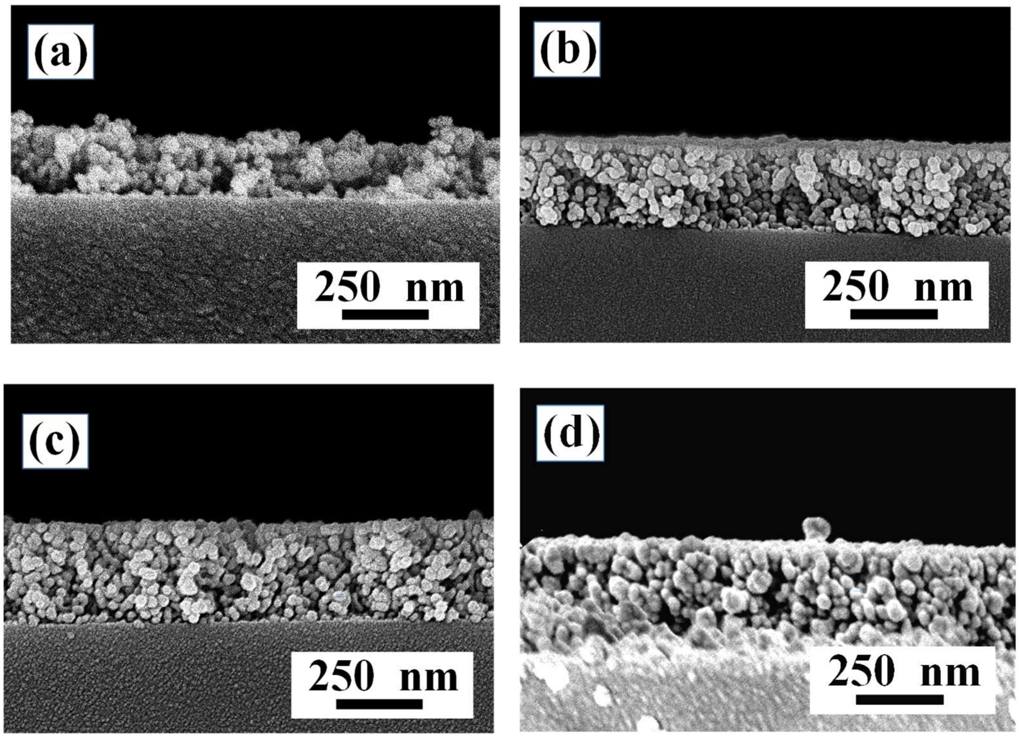

Figure S1 Cross-section SEM images of the L2NiO thin films as a function of annealing temperatures and times: (a) 400°C for 1 h, (b) 400°C for 3 h, (c) 500°C for 3 h, and (d) 600°C for 3 h.

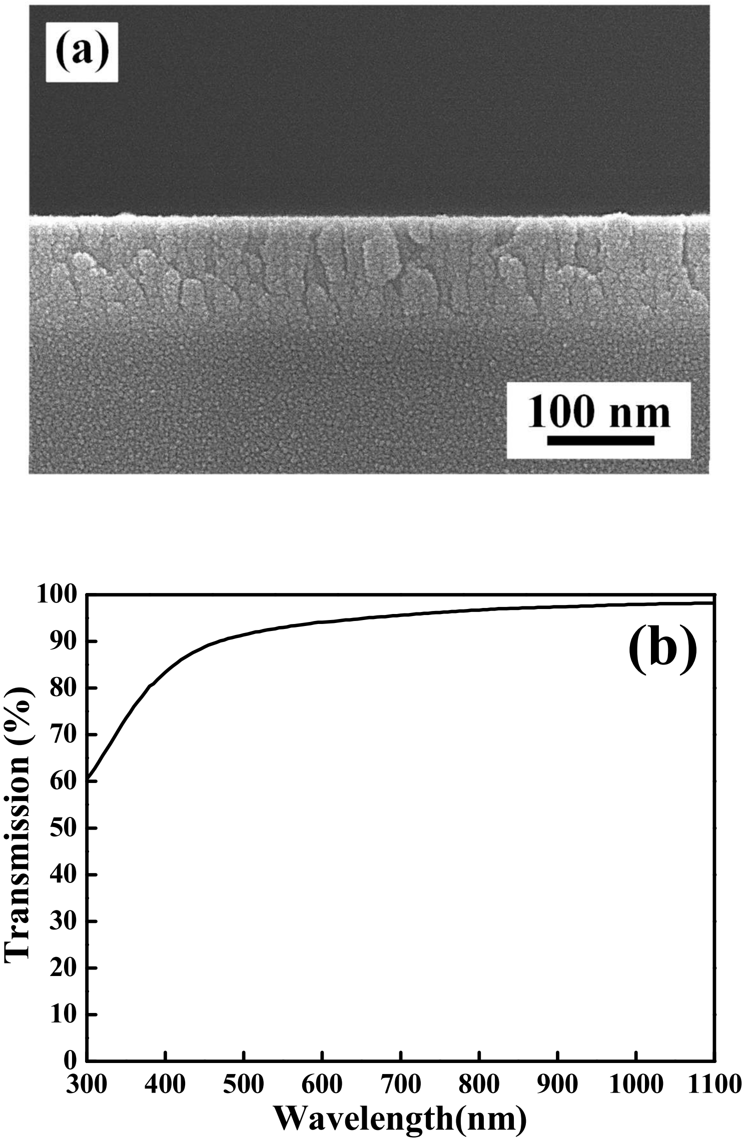

Figure S2 (a) Cross-section SEM image and (b) optical transmittance spectra of the ITO thin film.
